# The Efficacy of a Metacognitive Training Program in Amnestic Mild Cognitive Impairment: A 6-Month Follow-Up Clinical Study

**DOI:** 10.3390/healthcare12101019

**Published:** 2024-05-14

**Authors:** Grigoria Bampa, Despina Moraitou, Panagiota Metallidou, Elvira Masoura, Georgia Papantoniou, Maria Sofologi, Georgios A. Kougioumtzis, Magdalini Tsolaki

**Affiliations:** 1 Laboratory of Psychology, Department of Cognition, Brain and Behavior, School of Psychology, Aristotle University of Thessaloniki, 54124 Thessaloniki, Greece; demorait@psy.auth.gr (D.M.); pmetall@psy.auth.gr (P.M.); emasoura@psy.auth.gr (E.M.); 2 Laboratory of Neurodegenerative Diseases, Center of Interdisciplinary Research and Innovation (CIRI–AUTH), Balcan Center, Buildings A & B, 10th km Thessaloniki-Thermi, 54124 Thessaloniki, Greece; tsolakim1@gmail.com; 3 Laboratory of Psychology, Department of Early Childhood Education, School of Education, University of Ioannina, 45110 Ioannina, Greece; gpapanto@uoi.gr (G.P.); m.sofologi@uoi.gr (M.S.); 4 Institute of Humanities and Social Sciences, University Research Centre of Ioannina (URCI), 45110 Ioannina, Greece; 5 Department of Turkish and Modern Asian Studies, National and Kapodistrian University of Athens, 15772 Athens, Greece; gkougioum@ppp.uoa.gr; 6 Department of Psychology, Neapolis University Pafos, 8042 Pafos, Cyprus; 7 Greek Association of Alzheimer’s Disease and Related Disorders (GAADRD), 54643 Thessaloniki, Greece

**Keywords:** metacognitive training program, mild cognitive impairment, cognitive flexibility, metacognitive control, metacognitive beliefs, cognitive strategies

## Abstract

This study was conducted in response to the increasing prevalence of Alzheimer’s disease (AD) dementia and the significant risk faced by individuals with amnestic mild cognitive impairment with multiple-domain deficits (aMCI-md). Given the promising effects of MTPs, the primary aim of this study was to further explore their impact by assessing the maintenance of their benefits. Thus, 45 participants were randomly allocated in two groups: the Experimental group (*n* = 22), which received the metacognitive training program (MTP), and the Control group (n = 23) that received the cognitive exercises program (CEP). The training programs—the MTP and the CEP—included 10 individual sessions of a one-hour duration and took place once per week. To test the efficacy of the MTP, cognitive and metacognitive outcomes were compared between two groups—Experimental (EG) and Control (CG)—at four distinct time points: before–after–3 months–6 months after intervention. Based on this study’s findings, the positive effects of the MTP were evident over a six-month period. Specifically, already three months post-training, the CG began to show a decline in training-related gains. In contrast, the EG’s performance consistently improved, highlighting the superior efficacy of the MTP. Gains attributed to the MTP were detected in cognitive measures: cognitive flexibility and immediate visual recall, as well as in metacognitive measures: metacognitive control, improved metacognitive beliefs of attention, and an increased use of cognitive strategies. In conclusion, the results demonstrated the sustained effects of the MTP in cognitive and metacognitive measures over a period of six months, providing novel insight into the application and efficacy of the MTP in individuals with MCI.

## 1. Introduction

Mild cognitive impairment (MCI) represents a transitional stage in the cognitive decline continuum, where individuals exhibit noticeable cognitive impairments beyond those expected for their age and education level but do not meet the criteria for dementia [1]. MCI is generally divided into two primary subtypes: amnestic MCI (aMCI), which is mainly characterized by memory deterioration, and non-amnestic MCI, where memory is preserved, but other cognitive functions (such as language, attention, or visuospatial skills) may be impaired [1]. People with aMCI and particularly those with multiple deficits beyond memory ones (aMCI-md) have an increased risk of progressing to Alzheimer’s disease (AD) [2,3]. The impact of MCI on a person’s life is multifaceted. The struggle with cognitive challenges often leads to the emergence of anxiety and depressive symptoms. These emotional disturbances can significantly affect individuals’ relationships and social life, thereby adversely impacting their overall quality of life [4,5]. Hence, the need for effective interventions to delay the course of cognitive deterioration but also to enhance the overall quality of life is of primary importance. 

Several reviews and meta-analyses have been conducted in order to evaluate the efficacy of cognitive training programs designed for this population [6,7,8,9,10,11,12]. The range of these programs is diverse, including both domain-specific and multiple-domain training programs (targeting various aspects like cognition, nutrition, physical activity, etc.). While results support the efficacy of multi-domain training with modest effects on cognitive performance and more significant effects on well-being [6,7,8,9], there is also an increasing interest in modern technology-based interventions. These include computerized [11] and virtual reality training programs [12,13] which have demonstrated promising effects on cognitive performance. However, questions remain regarding their generalizability and the long-term maintenance of effects.

Over the past few decades, there has been an increased scientific interest in metacognition and its role in MCI. This trend is driven by the critical role metacognition plays in controlling and regulating cognitive processes [14]. Metacognitive regulation draws from an individual’s accumulated knowledge about cognitive principles and their personal appraisals of their cognitive skills (metacognitive knowledge) [14,15,16]. It is also influenced by monitoring processes that mirror ongoing cognitive activities (metacognitive monitoring) and assist with decision-making and adjustments in support of these cognitive processes (metacognitive control) [15,17,18,19].

Findings have revealed that individuals with MCI are fairly accurate when assessing their general cognitive status [20,21,22]. Yet, compared to cognitively healthy older adults, they display less accuracy when monitoring their performance during task engagement [23,24,25,26]. Accordingly, in a previous work by our team, we found that people with MCI although they were aware of their struggles during task engagement, as reflected by their confidence ratings, they were less accurate in their decisions to choose between wrong and right responses compared to cognitively healthy individuals [27]. Further, previous evidence about the difficulties that people with MCI have with prospective and not retrospective memory self-monitoring abilities [23] shows the need for training specific aspects of metamemory in MCI. Specifically, accurate Feeling of Knowing (FOK) ratings for future memory performance seem to be important not only for trying to access memory material but for employing memory strategies as well. FOK accuracy was found to be an important metamemory ability for episodic memory performance not only in individuals with MCI but in patients with Parkinson’s disease with akinetic and rigidity-dominant motor symptoms as well [28].

Accurate monitoring has a significant impact on cognitive performance. As previous studies have highlighted, the over/underestimation of one’s cognitive skills has negative consequences on the management of their deficits. Such inaccuracies can influence their motivation to undertake cognitively challenging situations, the effort they invest, and their application of compensatory strategies [29,30,31]. However, the ability to accurately appraise their cognitive performance does not translate to effective deficit management for individuals with MCI. Studies have demonstrated that even though individuals with MCI may gain some degree of monitoring accuracy and acknowledge their cognitive difficulties, they often lack the necessary skills or knowledge to properly manage these difficulties [31,32,33]. Similarly, based on a previous work by our team, it was found that individuals with MCI scored lower in metacognitive beliefs of efficacy particularly in daily life scenarios involving memory and attention (divided and shifted). Despite these lower scores, they did not report using cognitive strategies more frequently than individuals with intact cognitive status [34]. Consequently, they need to acquire explicit knowledge on how to improve self-regulatory skills like the effective allocation of time, the control of attention, the proper use of strategies or external aids, effort and time investment to learn new information, etc. [35,36,37,38]. This underscores the significance of examining the impact of cognitive training programs based on metacognitive principles, in individuals with MCI.

Hertzog et al. [39] presented a compelling approach that focuses on self-regulation with regards to everyday memory. Their findings emphasize the importance of personalized interventions, wherein everyone’s self-regulation skills are assessed to identify specific needs. Furthermore, the intervention should teach memory strategies and provide explicit knowledge on how, why, and when to utilize them effectively. Adequate feedback and encouragement are critical components to promote the successful transfer and application of these memory skills to everyday life. By adhering to these guidelines, memory enhancement interventions can have a more profound impact on older adults’ cognitive abilities, leading to improved overall well-being and autonomy.

Cognitive training programs that incorporate metacognitive aspects have been implemented across various populations, yielding significant benefits. In a systematic review conducted by our team [40], we investigated the effects of metacognitive training programs (MTPs) on adults, including older individuals and those with neurological conditions presenting a total of nine studies that utilized a randomized controlled design (RCT). Regarding older adults, existing research has shown that MTPs focusing on metamemory and the utilization of cognitive strategies can provide substantial benefits, including an enhanced understanding of how, why, and when to deploy cognitive strategies, along with improved self-regulatory processes, such as self-testing and study time allocation [33,35]. Furthermore, the role of self-efficacy—an individual’s confidence in their ability to perform cognitive tasks [41]—in learning how to effectively cope with cognitive difficulties cannot be overstated. Enhancing self-efficacy can motivate older adults to learn and apply more advanced cognitive strategies and improve their overall well-being [42,43]. Additional evidence supporting the metacognitive approach’s effectiveness comes from a new meta-analysis conducted by Sella et al. (2023) on metacognitive interventions in older adults. Overall, the findings showed improvements in cognitive performance, particularly in memory, as well as positive effects on memory strategy use and subjective beliefs about memory efficacy. However, the long-term effects of MTPs still require more in-depth investigation. Future studies should incorporate follow-ups to determine whether the observed training-related gains persist over time [40,44].

Available studies testing the efficacy of cognitive training programs with metacognitive components in individuals with MCI are very limited, though they have demonstrated potential benefits. Moro et al. [45] conducted a study assessing the effects of a personalized cognitive training program focusing on executive functions’ enhancement. The training lasted six months and included metacognitive components such as metacognitive knowledge about cognitive strategies and bolstering metacognitive monitoring and regulation during homework assignments. The findings revealed significant improvements in memory and overall cognitive status, which lasted over a period of six months. Likewise, Youn et al. [46] applied a ten-session metamemory training (MMT) program using an RCT design in participants with MCI. The results showed positive effects of MMT on verbal memory and verbal fluency performance, along with a reduction in subjective memory complaints compared to a passive control group (waiting list). To our knowledge, this is the only study that tested the efficacy of an MTP designed for MCI using an RCT design. Nevertheless, no follow-up assessments were conducted to test the maintenance of these gains over time.

In conclusion, people with MCI face cognitive challenges that intrude upon their daily life, often leading to social and psychological distress and negatively impacting their overall well-being. Therefore, cognitive training programs need to be multidimensional, addressing all these areas of concern. Although emerging evidence highlights the potential of metacognitive-based training programs to address these diverse needs, studies focusing specifically on this population are limited. Moreover, an important question that remains to be addressed is the sustainability of these benefits over time. Hence, the present study aimed to assess the efficacy of a ten-session MTP for adults with aMCI-md over a period of six months, using an RCT design.

### Aim and Hypotheses

This study was conducted in response to the increasing prevalence of AD dementia and the significant risk faced by individuals with aMCI-md. Given the promising effects of MTPs, the primary aim of this study was to further explore their impact by assessing the duration and maintenance of their benefits. Therefore, follow-up assessments were implemented at 3 and 6 months post-training. The impact of the MTP was tested on cognitive and metacognitive outcomes. To the best of our knowledge, this is the first study that investigates the effects of an MTP using online metacognitive measures. An RCT was employed that included an Experimental group (EG), which followed the metacognitive intervention, and an active Control group (CG), which followed a cognitive exercise program (CEP). 

Based on the expected benefits of metacognitive training, we anticipated that the advantages derived from metacognitive training in the EG would sustain over follow-up periods (3 and 6 months post-training), while any initially observed benefits in the CG were anticipated to fade over the same timeline.

## 2. Materials and Methods

### 2.1. Design

This study utilized a randomized longitudinal controlled design to compare two groups of MCI patients, matched in age, educational level, and gender: the Experimental group which followed the metacognitive intervention and the Control group which followed a cognitive intervention. Repeated measures were retrieved at four time points: (1) before the training (pre-training), (2) immediately after the training (post-training), (3) three months after the training (3-month follow-up), and (4) six months after the training (6-month follow-up). The main purpose of the repeated measures design was to assess whether metacognitive training vs. cognitive training can lead to longitudinal gains in terms of cognitive functioning improvement in MCI patients. 

### 2.2. Participants

Based on a power analysis that was conducted, using G*Power [47], a total sample size of 62 participants was recommended to detect an effect size of *η*^2^ = 0.15, with an alpha of 0.05 and to achieve a power of 0.80. In the current study, 50 aMCI patients agreed to participate in the training program. They were randomly divided into two equally sized groups, EG and CG, each containing 25 participants. However, attrition resulted in the completion of training sessions by 45 participants, as 5 individuals dropped out (3 from the EG and 2 from the CG). However, due to the prolonged lockdown measures taken to suppress COVID-19, it was not feasible to enroll more participants. Consequently, our final sample comprised 15 men and 30 women with a mean age of 62.78 (SD = 6.24) years and a mean education of 13.11 (SD = 3.45) years. To participate in the study, individuals were required to be native Greek speakers, over the age of 50, and have a minimum of six years of education. They underwent an extended neuropsychological assessment in accordance with Petersen’s diagnostic criteria [3] and DSM-V [48].

The neuropsychological evaluation was conducted at the Greek Association of Alzheimer’s Disease and Related Disorders by trained psychologists and included several tools including clinical scales and neuropsychological tests. Clinical Tools: the Geriatric Depression Scale [49,50], the Beck Depression Inventory [51], the Beck Anxiety Inventory [52], the Short Anxiety Screening Test [53,54], and the Neuropsychiatric Inventory [55,56] were used to test for affective disorders and/or neuropsychiatric symptoms. Cognitive tools: for general cognitive status screening, the Mini Mental State Examination [57,58] and the Montreal Cognitive Assessment [59,60] were implemented. The Functional Cognitive Assessment [61] was used to measure executive functions in six daily activities. Standardized cognitive tests were also performed to assess memory, attention, executive functions, and language skills. Tsolaki et al. [62] provide a detailed description of all the neuropsychological tests employed. The Global Deterioration Scale (GDS) [63] was used to define the stage of the participants’ cognitive decline. Thus, based on the GDS, participants with no cognitive decline or impairments were assigned stage 1, while those with mild cognitive impairment (MCI) were designated stage 3. The present study was particularly focused on participants who displayed the amnestic subtype of MCI characterized by multiple deficits. Hence, if an individual’s memory and one or more additional cognitive domains were observed to be significantly below average (1.5 standard deviations below their age norm), they were classified as having aMCI-md [64]. 

The exclusion criteria were as follows: (a) a history of psychiatric disorder; (b) substance abuse or alcoholism; (c) a history of traumatic brain injury; (d) a history of neurological disorders (brain tumor, epilepsy, encephalitis, Parkinson’s disease, multiple sclerosis); (e) diabetes (type I and II); (f) cardiovascular diseases; (g) sensorimotor deficits that could interfere with study procedures; (h) vitamin B12 deficiency; and (i) a lack of memory deficits (naMCI). 

### 2.3. Procedure

Participants were recruited from the “Agia Eleni” day care center of the Greek Association of Alzheimer’s Disease and Related Disorders and via Aristotle University of Thessaloniki. The recruitment process was assisted by undergraduate psychology students as part of their clinical internships. Potential participants who met the study’s eligibility criteria were invited to volunteer. 

Upon receiving consent, the study’s neuropsychologist (first author) assessed the eligibility of each participant, providing them with an overview of the study, its objectives, and the procedures involved. Participants were informed that the testing process would be divided into two separate morning sessions, designed to eliminate the effects of fatigue. Each session was limited to one hour. During the initial meeting, participants were presented with written consent forms that explicitly stated the study’s objectives, assuring them of the strict confidentiality and privacy of their personal data in accordance with data protection guidelines. Participants were also informed about a potential 10-session training program that would be arranged at their convenience after the initial testing sessions, which would be followed by two post-training assessments (at three and six months after training’s completion) following the same format as the initial sessions. The participants did not receive details about the specific content of each training program, adhering to a single-blind procedure.

Due to extended lockdown restrictions for COVID-19 prevention, all procedures were conducted virtually through online platforms such as Skype, Zoom, Messenger, and Viber. As such, only participants with access to desktops, laptops, or tablets were eligible for this study, as mobile phones were deemed unsuitable due to their small screens. Participants in the EG were also asked to provide an email address, either their own or a relative’s, in order to receive the training-related materials. This information would be treated with the same strict confidentiality and privacy measures and used solely for the purpose of the study.

In addressing external factors, participants were instructed to be in a quiet room without external distractions, ensuring no other individuals were present and that televisions were turned off while phones were set to silent mode. These arrangements were thoroughly discussed with participants in advance of the testing procedure and programs’ initiation, with provisions made for assistance from a relative if needed. While the control of these external factors ultimately rested with the participants, it was assumed that they would adhere to the prescribed arrangements. Overall, compliance with these guidelines was largely observed, with only minor deviations noted.

### 2.4. Cognitive Measures

#### 2.4.1. Wisconsin Card Sorting Test—64 Card Version (WCST-64)

The WCST-64 [65] is a compact version of the original test [66,67], consisting of 64 sorting cards based on color, shape, and number, compared to the original’s 128 cards. This test evaluates cognitive flexibility, the ability to switch cognitive sets, and the capacity to utilize feedback in problem-solving tasks. Despite its abbreviated length, the WCST-64 preserves the principal structure and administration guidelines of the original, prompting participants to match cards according to undisclosed, changing rules and offering feedback to influence their responses. The WCST-64 maintains robust psychometric characteristics, such as significant test–retest reliability [68,69,70] and construct validity, demonstrated by its ability to identify frontal lobe dysfunction [71] and its correlations with other executive functioning measures [72]. The WCST-64 serves as an efficient alternative to the full-length WCST, maintaining its diagnostic worth and adaptability across different population groups and clinical settings [73].

Some of the key scores derived from the WCST include the following [74]: (1) Total Correct: The total number of correct responses given by the participant throughout the test. A higher score indicates better performance and cognitive flexibility; (2) Total Errors: The total number of incorrect responses given by the participant throughout the test. A lower score indicates better performance and fewer mistakes made; (3) Perseverative Responses: The number of times the participant sticks to a previously correct sorting rule even after it has become invalid. A lower score indicates better cognitive adaptability and readiness to alter rules; (4) Perseverative Errors: The number of errors made by the participant due to continuously applying an incorrect rule or strategy, even after receiving feedback that it is no longer applicable. A lower score indicates better cognitive adaptability and responsiveness to new information; (5) Non-Perseverative Errors: The number of incorrect responses that are not perseverative in nature. A lower score indicates better performance in terms of adaptability and problem-solving; (6) Categories Completed: The number of categories (out of a possible six) that the participant successfully completed during the test. A higher score indicates better cognitive flexibility and abstract reasoning; (7) Trials to Complete First Category: The number of trials needed for the participant to successfully complete the first category. A lower score indicates a quicker understanding of the sorting rules and more efficient problem-solving skills; and (8) Failure to Maintain Set: The number of times the participant failed to maintain a correct sorting rule after successfully applying it for a few consecutive trials. A lower score indicates better cognitive stability and consistency in applying learned rules.

#### 2.4.2. Doors and People 

Doors and People [75] is a test designed to assess memory function and includes four subtests, each of which assesses different aspects of memory: People, Doors, Figures, and Names. This test has been adapted and validated in the Greek population [76]. Ιt is a reliable tool with ecological validity and satisfactory internal validity (Cronbach’s α = 0.80).

The People subtest evaluates verbal recall, both immediate (three trials) and delayed, by presenting a list of names and later asking the participant to recall them. The stimuli specifically consist of photos of four individuals, with their names and professions displayed below. Each picture is displayed for 3 s, and the character’s name and job are spoken aloud [e.g., This is a doctor. His name is Hλίας Τσακίρης (Elias Tsakiris)]. This process is carried out until all four names are accurately remembered (with a maximum of three attempts). Participants are then asked to recall this information immediately after the presentation and again after a 5–10 min delay. A single point is awarded for each correctly remembered first and last name, with an additional point given for each correct pairing. The total score is computed by adding the scores from each trial (score range: 0–36) [77]. 

The Doors subtest evaluates visual recognition by presenting images of doors and subsequently asking the participant to pick out the previously viewed doors from a set of new ones. Specifically, participants are exposed to 24 images of doors, divided into two sets (one easy set and one hard set). Following the presentation, they are asked to identify the previously seen door from a set of four doors (three distractors and the target door). In the first set (Part A), the distractors are varied types of doors in comparison to the target door (for instance, a garage door, a German door, a front door), whereas in the second set (Part B), the distractors are from the same category of doors as the target (for example, all stable doors). One point is given for each correct answer, and the total score is computed by adding the scores in each set (score range: 0–24) [77].

The Figures subtest evaluates immediate and delayed visual recall. This is accomplished by presenting a set of figures to the participants and subsequently asking them to draw as many as they can recall. Participants are shown four line-drawn crosses and asked to draw them immediately after the presentation and again after a 5–10 min delay. The figures are presented until the participant can accurately reproduce them (with a maximum of three attempts). Each accurately drawn shape earns three points, and the total score is computed by adding the scores from each trial (score range: 0–36) [77].

Finally, the Names subtest evaluates verbal recognition by displaying a list of names and then asking the participant to recall which names were shown previously. Participants are given twenty-four names (including both first and last names), separated into two groups (an easy set and a hard set). Each name is presented for 3 s, and participants are instructed to read them out loud. After the presentation, they are asked to identify the previously shown name from a set of four names (three distractors and the target name). The second set (Part B) consists of names where distractors closely resemble the target name. One point is given for each correct identification, and the total score is computed by adding the scores in each set (score range: 0–24) [77].

### 2.5. Metacognitive Measures

For the aim of the present study, the two cognitive tests—the Wisconsin Card Sorting Test and the Doors and People test—were used in a metacognitive version [78,79]. Following each response, participants were instructed to answer two questions: (1) “What is your degree of confidence in the correctness of this answer?” (feeling of confidence appraisals), and (2) “Would you like your response to be included in the total score?” (decisions on whether to volunteer or not a response to maximize final score performance as a measure of metacognitive control). Participants replied on a 4-point Likert scale (1 = not at all certain, 4 = completely certain) for the first question and on a Yes/No format for the second question. It was specified that a correct “yes” answer would increase their score by one point, an incorrect “yes” answer would subtract a point, and a “no” answer, regardless of its accuracy, would leave their score unchanged.

Based on the two metacognitive questions, four metacognitive variables were extracted: (1) the relative confidence ratings (on a scale from 1 to 4), (2) the accuracy score (calculated as the ratio of correct volunteered responses, i.e., correct “yes,” compared to total volunteered responses, i.e., total “yes.” This variable reflects the reliability of a person’s responses and depends on monitoring and control procedures), (3) global monitoring (this refers to an individual’s ability to estimate their overall performance on a task. It was calculated as the difference between the total correct responses, i.e., actual performance, and the total volunteered responses, i.e., total “yes.” Scores below zero indicate overconfidence, while scores above zero suggest underconfidence), and (4) wrong “yes” (the quantity of incorrect volunteered responses, where fewer numbers suggest a more careful decision-making approach, and larger numbers indicate a riskier approach).

Metacognitive ability was determined using the ratio of the mean feeling of confidence to the cognitive score. This ratio establishes the relationship between reported confidence and actual performance. The feeling of confidence ranges from 1 (lowest) to 4 (highest), whereas the cognitive score (the number of correct responses divided by total items) ranges from 0 (no correct answers) to 1 (all answers correct). Thus, a calibration score of 4 signifies perfect alignment between confidence and performance. Scores below 4 suggest underconfidence, while scores above 4 indicate overconfidence.

#### 2.5.1. Metacognitive Knowledge for Everyday Memory (MKEM)

MKEM [80] is a 12-item self-report scale developed to assess older adults’ metacognitive beliefs about everyday life scenarios related to memory function. For each scenario, participants were requested to report their degree of efficacy on a 4-point Likert scale, ranging from 1 “not at all” to 4 “very well” (example: Imagine that you want to tell a story that you read earlier in a book or in a newspaper. How well do you manage to remember the details of that story, such as names, the place, and time?). The scale has a one-factor structure and high internal reliability (α = 0.88).

#### 2.5.2. Metacognitive Knowledge for Everyday Attention (MKEA)

MKEA [80] is a 12-item self-report scale developed to assess older adults’ metacognitive beliefs about everyday life scenarios related to attention. For each scenario, participants were requested to report their degree of efficacy on a 4-point Likert scale, ranging from 1 “not at all” to 4 “very well” (example: Imagine that you are at the bank, and you are waiting for your number to appear on the announcement table. How well do you manage to stay focused so that you do not lose your turn when your number appears?). The scale has a two-factor structure, with factors reflecting “Divided and Shifted Attention” (α = 0.74) and “Concentration” (α = 0.75).

#### 2.5.3. Metacognitive Knowledge for Everyday Executive Functions (MKEEFs)

MKEEFs [80] is a 10-item self-report scale developed to assess older adults’ metacognitive beliefs about everyday life scenarios related to executive functions. For each scenario, participants were requested to report their degree of efficacy on a 4-point Likert scale, ranging from 1 “not at all” to 4 “very well” (example: Imagine that you have planned to go on a walk with a friend, but it starts raining. How well do you manage to think of an alternative plan considering the weather (i.e., sit in a cafeteria)?). The scale has a two-factor structure, with factors reflecting “Planning” (α = 0.70) and “Inhibition” (α = 0.65).

#### 2.5.4. Multifactorial Memory Questionnaire—Strategies Subscale (MMQ)

The MMQ [81] is a self-report scale comprising 57 items, developed to evaluate metamemory in older adults. It incorporates three subscales: (1) *Contentment*, which measures one’s sentiment towards their memory; (2) *Ability*, which measures an individual’s assessment of their memory skills; and (3) *Strategies*, which measures the frequency of an individual’s use of cognitive strategies. This tool exhibits robust psychometric characteristics. A Greek version of the scale, translated and slightly modified, is made available by Evdokia Emmanouilidou, Nikoleta Fratzi, and Despina Moraitou. The document can be found at https://www.baycrest.org/Baycrest_Centre/media/content/MMQ-Greek.pdf (accessed on 10 January 2023). For the purposes of the present study, we only used the Strategies subscale, which is a 19-item self-report questionnaire. The responses were given on a 5-point Likert scale ranging from 0 (never) to 4 (always) with higher scores indicating a more frequent use of strategies (example: How often do you make a list, such as a grocery list or a list of things to do?). 

The scale has a two-factor structure (structural validity and internal reliability previously tested by our team using the responses of 100 participants). The MMQ—Strategies subscale was assessed in a previous work of ours, and a two-factor solution emerged as the best fit. The first factor “Simple Strategies” (α = 0.78) reflected the utilization of external aids or the implementation of simple cognitive regulatory processes, such as organizing information. The second factor “Complex Strategies” (α = 0.73) reflected more intricate and demanding cognitive strategies that necessitate greater effort and involve complex information processing, such as mental imagery and story creation. 

### 2.6. Interventions

#### 2.6.1. Metacognitive Training Program (MTP)

The content and structure of the MTP were designed based on existing research concerning metacognition and aging, as well as on training programs specifically targeted at improving metacognition in older adults [35,37,38,82]. Recognizing the documented deficits in metacognitive accuracy and the need for effective cognitive strategies in MCI, we developed a program aimed at enhancing metacognitive knowledge and the processes of metacognitive monitoring and control [40,44]. Moreover, given that MCI, particularly aMCI-md, impacts not just memory but also other cognitive domains, our MTP does not solely focus on memory [1]. It includes information and practical exercises related to attention and executive functions. Despite the impairment in other cognitive domains, it is vital for participants to gain knowledge about these areas to identify their own cognitive strengths and weaknesses, considering the interrelated and non-independent nature of cognitive functions. Before launching the study, the MTP was pilot-tested with five volunteers diagnosed with MCI to address practical considerations such as session duration, the clarity and comprehension of the content and exercises, the number of exercises, and the level of difficulty. The feedback from this pilot application was used to make the necessary adjustments to the program.

The MTP included 10 individual sessions that were carried out on a weekly basis. The duration of each session was approximately one hour. The context of each session varied including the following topics: memory, attention, executive functions, and cognitive strategies (see Table 1 for the detailed presentation of each session). Regarding cognitive strategies, four sessions were conducted in order to gain practical experience and guidance on how to use them in different tasks in daily life. Thus, an introductory session was conducted in which various strategies were presented and discussed, emphasizing the importance of using such strategies. Furthermore, the significance of evaluating a strategy’s effectiveness and making necessary adjustments was highlighted based on self-testing [35,37]. Then, three sessions followed focusing on the application and practice of four specific strategies: mental imagery, story creation, paired association (verbal and visual), and categorization. These strategies were chosen based on existing scientific evidence supporting their effectiveness and their applicability across various contexts and types of information [83,84,85,86].

The training material was delivered through PowerPoint presentations, employing clear, simple language while still including specific terminology such as episodic memory, short-term memory, and executive functions. This approach allowed for a comprehensive coverage of the topic at hand. The presentations were shared with participants through screen-sharing, allowing the study’s neuropsychologist to simultaneously explain the relevant material. Participants were actively encouraged to engage with the content by asking questions and sharing personal experiences related to the material. This interactive approach helps to promote a deeper understanding of the various concepts being discussed [84]. Through this methodology, we ensured that participants were not only recipients of knowledge but also active contributors to the learning process. 

Between the sessions, participants were provided with homework assignments relevant to the topic of the preceding session, which were delivered via email. The purpose of these assignments was to reinforce metacognitive monitoring and regulatory skills through practical exercises [35,45,84]. At the beginning of each subsequent session, time was allocated to review the completed assignments, address any remaining questions, and facilitate a thorough understanding of the material. Then, the material of the new session was presented and discussed. At the end of each session, the new homework assignments were presented and explained to ensure that participants understood what they have to do. 

The homework assignments were carefully designed to reflect the topic of each session and to integrate real-world tasks. For example, participants might be given a list of words or a story to learn and later recall, as a homework assignment related to the session on memory. In the session on attention, they might be tasked with counting how many advertisements are shown during a TV program. In the session addressing executive functions, participants were given a map of Athens’ public transportation and were instructed to navigate it to solve a series of tasks. To give an example, “Imagine you are in Ampelokipoi and you want to go to Larissa Station. However, the M2 metro line (red line) is closed from Neos Kosmos to Metaxourgio. Describe what you would do to reach your destination.”, and so on. These exercises incorporated real-world tasks to make the training as practical and applicable to daily life as possible.

To reinforce self-monitoring and self-regulation, after each exercise, participants were instructed to self-test by noting down their score and responding to certain questions [35,45]. Examples of these questions include the following:

1. “How easy was it for you to maintain your attention on the task at hand?”
2. “Did you had any difficulties in maintaining your attention? If yes, what was it?”
3. “Write down what you did in order to learn the story.”
4. “Of the two strategies you used, which one was more effective?”
5. “Think and describe other situations in which you could use the strategy that helped you the most. Provide 2 or 3 examples.”

#### 2.6.2. Cognitive Exercises Program (CEP)

A training program of equal duration, also conducted through individual sessions, was provided to the CG. In this program, participants were given a variety of vocabulary and verbal fluency exercises for cognitive practice. These exercises were displayed via screen-sharing. Instructions were clearly explained, and at the end of each session, participants were provided with the correct answers to cross-check their responses. Participants were advised to have paper and a pen/pencil ready to solve the exercises. Unlike the MTP, the program provided to the CG did not involve any discussion about cognitive functions, age-related deficits, or the use of strategies. Their sessions were purely focused on completing cognitive exercises.

### 2.7. Statistical Analysis

The statistical analysis was conducted using IBM SPSS Statistics Version 27 (IBM Corp. Released 2020. IBM SPSS Statistics for Macintosh, Version 27.0. Armonk, NY: IBM Corp). The analyses carried out were a (a) mixed measures ANOVA and (b) Kruskal–Wallis non-parametric test. The aim of these analyses was to compare the performance between the two groups as well as the performance of each group at different time points. The Partial eta-squared (η^2^) measure was used to estimate the effect size. The threshold for statistical significance was set at *p* < 0.05.

### 2.8. Ethics

This study’s purpose was both verbally and in written form communicated to the participants, and they were assured that their data would be kept confidential. They gave their written consent, acknowledging their participation was voluntary, and they could opt out at any moment. Demographic information, such as age, gender, and education, was collected, adhering to the law of the European Union since 28 May 2018, which allows the use of sensitive personal data for research purposes. Participants were told and consented to that, upon a written request, their data could be removed from the online database. The research protocol was approved by the Scientific and Ethics Committee of the Greek Association of Alzheimer’s Disease and Related Disorders (Approval Code: 29/15-02-2017), in compliance with the guidelines of the Helsinki Declaration.

## 3. Results

A univariate analysis of variance (ANOVA) was conducted to examine whether the two groups differed in age (in years) and years of education. The statistical analysis revealed no significant differences between the two groups for age, F(1, 43) = 0.26, *p* = 0.61, or for years of education, F(1, 43) = 0.83, *p* = 0.37. In addition, a chi-square analysis regarding gender distribution showed that there were also no statistically significant differences between the groups, χ^2^ (1) = 0.18, *p* = 0.67. Therefore, the two groups were matched in age, education, and gender distribution (see Table 2). 

Mixed-design 4 × 2 ANOVAs (representing four time measurements × two groups, EG, CG) were conducted to examine the impact of the metacognitive training program on each of the cognitive performance variables and metacognitive outcomes. The study involved a total of 45 participants. However, four participants (two from each group) did not complete the post-training phase. Also, one participant from the CG withdrew after the post-training measurement for health reasons. The scores of these individuals were excluded in the ANOVAs. Thus, the mixed-design ANOVA analyses were conducted with 39 participants.

### 3.1. Effects of Metacognitive Training on Cognitive Performance

Cognitive performance was assessed through eight variables for the WCST: total correct responses, total errors, perseverative errors, non-perseverative errors, perseverative responses, categories completed, trials to complete the first category, and failure to maintain category, along with six variables for the Doors and People test: people, doors, figures, names, delayed verbal recall, and delayed visual recall. The within-subjects factor in all the mixed-design ANOVAs was the measurement time point, with four levels: pre-training, post-training, and follow-ups at 3 and 6 months; while the between-subjects factor was the group, with two levels: Experimental and Control groups (EG, CG). 

**Figure 1:**
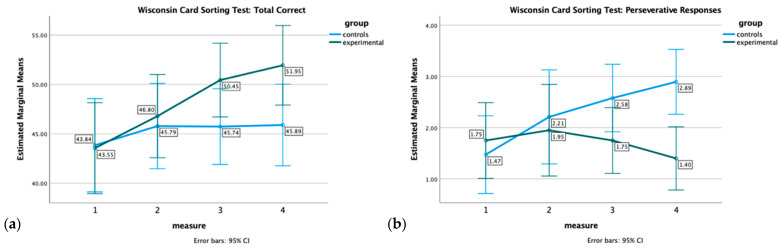
The figure displays the performance of the Experimental and Control group on (**a**) the WCST: total correct and (**b**) WCST: perseverative responses at four time points.

For the WCST scores, the results revealed a significant main effect of measurement on the total correct responses score, F(1.96, 72.38) = 7.79, *p* = 0.002, η^2^ = 0.15, total errors, F(1.96, 72.33) = 7.87, *p* < 0.001, η^2^ = 0.18, non-perseverative errors, F(1.84, 67.98) = 15.19, *p* < 0.001, η^2^ = 0.29, and categories completed, F(2.39, 88.53) = 4.85, *p* = 0.007, η^2^ = 0.12, indicating improvement in these variables across the four time points. However, the main effect of the group and the measurement x group interaction were not significant for these variables, suggesting no significant differences between the two groups. However, as one can indicatively see in Figure 1, a clear trend emerges in the third and fourth measurement. Specifically, in Figure 1a, one can observe consistent improvement in the EG’s performance over time, whereas the CG’s performance stays stable. In Figure 1b, the performance of the EG continues to rise as the number of perseverative responses decreases, while the CG’s performance consistently declines as the number of perseverative responses grows. These observations suggest lasting effects of the MTP.

**Figure 2:**
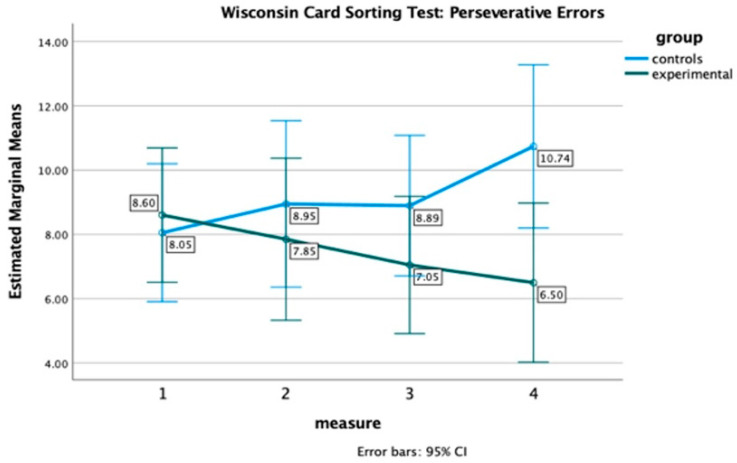
Performance of Experimental and Control group on WCST: perseverative errors at four time points.

A significant interaction effect was only found on perseverative errors, F(2.40, 88.85) = 3.54, *p* = 0.03, η^2^ = 0.09, suggesting that the patterns of change in perseverative errors over time differed between the two groups. However, the effect size was very small (η^2^ = 0.09), and no significant main effect was found for measurement, F(2.40, 88.85) = 0.26, *p* = 0.81, or group, F(1, 37) = 1.50, *p* = 0.23. Yet, as shown in Figure 2, the EG’s performance consistently improved over time by decreasing perseverative errors, while the CG exhibits a progressive decline (as increasing errors) in performance on this measure. Again, the discrepancy between the two groups becomes more noticeable at the 6-month follow-up. 

Bonferroni pairwise comparisons revealed that both—Experimental and Control— groups performed better at the 6-month follow-up compared to baseline (pre-training) in the following scores: total correct, total errors, non-perseverative errors, and categories (a detailed presentation of these results can be found in Table 3).

**Figure 3:**
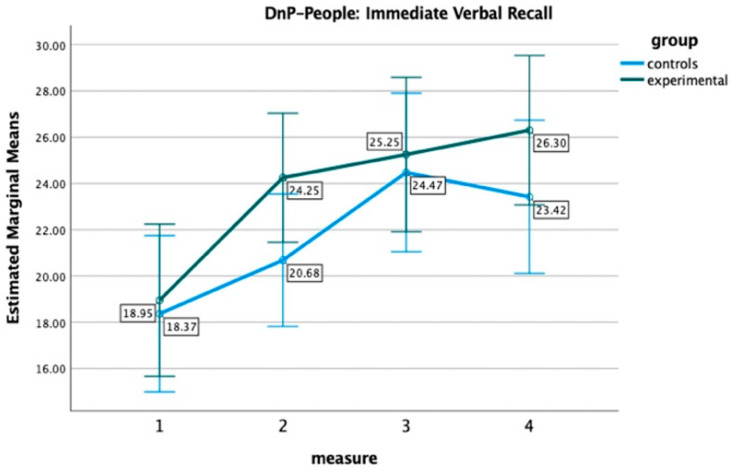
Performance of Experimental and Control group on immediate verbal recall at four time points.

Moving on to the Doors and People scores, the results from the respective mixed-measures ANOVAs revealed a significant main effect of the time of measurement on people, F(2.67, 98.68) = 17.60, *p* < 0.001, η^2^ = 0.32, doors, F(2.88, 106.69) = 22.96, *p* < 0.001, η^2^ = 0.38, figures, F(2.05, 67.98) = 6.57, *p* = 0.002, η^2^ = 0.15, names, F(2.61, 96.50) = 9.12, *p* < 0.001, η^2^ = 0.20, and delayed verbal recall, F(2.76, 102.05) = 8.21, *p* < 0.001, η^2^ = 0.18, indicating an improvement in these variables across the four time points. A main effect of group or an interaction effect (measurement x group) was not detected in all these analyses, suggesting no significant differences between the two groups. However, once again, Figure 3 indicatively illustrates a trend favoring the EG in immediate verbal recall. Specifically, the EG’s ascending performance persists at the 6-month follow-up, while the CG begins to exhibit a decline after the 3-month follow-up.

Pairwise comparisons revealed that both—Experimental and Control—groups performed better after the interventions (at post-training, at 3-month follow-up, and at 6-month follow-up) compared to baseline (pre-training) in immediate verbal (People) and visual (Figures) recall and in visual recognition (Doors). Also, significant improvements for the two groups were detected in verbal recognition (Names) and in delayed verbal recall (People-Delayed) at the 3- and 6-month follow-up compared to pre-training (a detailed presentation of these results can be found in Table 3).

### 3.2. Effects of Metacognitive Training on Metacognitive Outcomes

Metacognition was assessed through four online metacognitive variables: monitoring accuracy, global monitoring, wrong yes, and the feeling of confidence, along with seven offline metacognitive variables: MMQ—Complex Strategies; MMQ—Simple Strategies; MKEM; MKEA—Divided and Shifted Attention; MKEA—Concentration; MKEEFs—Inhibition; and MKEEFs—Planning. For each time point, a mean score for each aspect of the offline metacognitive measures was calculated. Also, an average score was calculated for each online metacognitive measure (monitoring accuracy, global monitoring, ‘wrong yes’, and the feeling of confidence). Thus, each resulting score represents the mean value across all tasks at that time point, providing an overall view of participants’ metacognitive performance over time. Again, as regards the mixed measures ANOVAs, the within-subjects factor was the measurement time point, with four levels: pre-training, post-training, and follow-ups at 3 and 6 months; while the between-subjects factor was the group, with two levels: Experimental and Control. 

**Figure 4:**
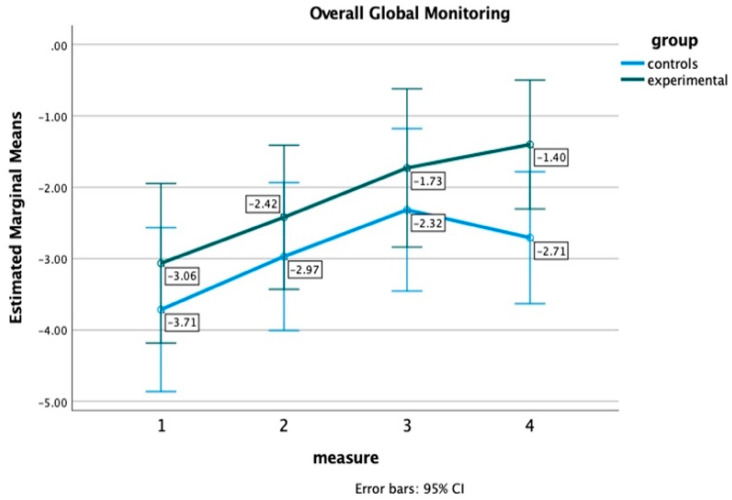
Overall global monitoring for the Experimental and Control group at four time points.

For the online metacognitive measures, the results revealed a significant main effect of the time of measurement on all three metacognitive control variables: monitoring accuracy, F(2.47, 91.33) = 10.77, *p* < 0.001, η^2^ = 23, global monitoring, F(2.24, 82.85) = 8.57, *p* < 0.001, η^2^ = 0.20, wrong yes, F(2.35, 86.90) = 20.01, *p* < 0.001, η^2^ = 0.35, and on metacognitive ability, F(2.15, 79.44) = 5.92, *p* = 0.003, η^2^ = 0.14, indicating an improvement in these variables across the four time points for both groups. A main effect of group or an interaction effect (measurement x group) was not detected, suggesting no significant differences between the two groups. However, as indicatively shown in Figure 4, the EG’s ascending performance regarding overall global monitoring continues at the 6-month follow-up, while the CG begins to exhibit a decline three months after training, suggesting the long-term impact of the MTP on these measures as well.

**Figure 5:**
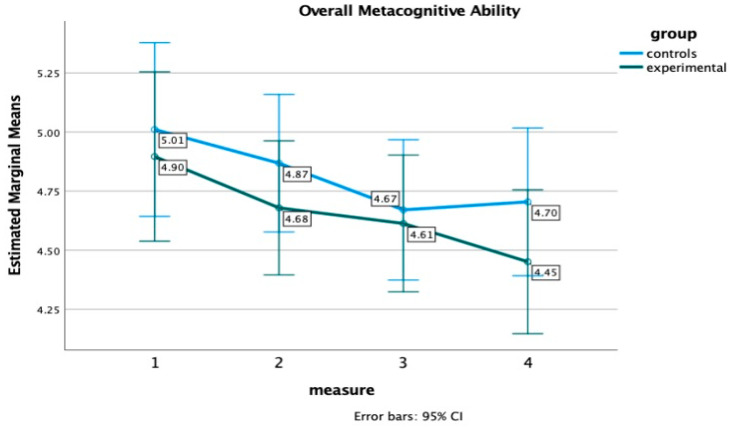
Overall metacognitive ability for the Experimental and Control group at four time points.

Likewise, as illustrated in Figure 5, participants exhibited an improved metacognitive ability score following the training programs, leading towards a better alignment between their confidence and actual performance; a trend emerges after the 3-month follow-up. Specifically, the EG showed a consistent pattern of improvement, gradually nearing a score of 4 which signifies a perfect alignment between confidence and actual performance, whereas the CG displayed a rising trend (above 4) indicative of increasing overconfidence.

Pairwise comparisons for online metacognitive measures revealed significant differences between pre-training and the subsequent three measures (post-training, 3-month follow-up, and 6-month follow-up) concerning metacognitive control measures. Additionally, significant variations were observed between the initial assessment and the 3-month as well as 6-month follow-up assessments for metacognitive ability. These results indicated that improvements occurred in both groups (Experimental and Control) following the interventions (a detailed presentation of these results can be found in Table 3).

Regarding the offline metacognitive measures, the results revealed a significant main effect of the time of measurement on MMQ—Complex Strategies, F(2.71, 100.11) = 10.23, *p* < 0.001, η^2^ = 0.22, and on MKEM, F(2.93, 108.66) = 4.31, *p* = 0.007, η^2^ = 0.10. These results indicate significant changes in these measures across the four time points. A main effect of group or an interaction effect (measurement x group) was not detected, suggesting no significant differences between the two groups. 

Pairwise comparisons for the MMQ—Complex Strategies showed that the EG and CG reported an increased use of complex strategies at post-training and at the 3-month and 6-month follow-up compared to pre-training, while for MKEM, they did not reveal any significant difference between specific time points (a detailed presentation of these results can be found in Table 3). 

Nevertheless, a significant main effect of the time of measurement, F(2.61, 96.56) = 3.61, *p* = 0.021, η^2^ = 0.09, and a significant measurement x group interaction effect, F(2.61, 96.56) = 4.57, *p* = 0.007, η^2^ = 0.11, were detected on MKEA—Divided and Shifted Attention, indicating that the patterns of change over time varied between the two groups. However, no significant main effect of the group variable was detected, F(1, 37) = 0.48, *p* = 0.50. Also, pairwise comparisons did not reveal any significant result between specific time points. 

Given the inherent problem with power in the study, and the small n of participants in each group as well as the clear trends as described in the figures, it becomes obvious that we were not able to detect what exactly happens in the performance of each group in the third and fourth measurements (for additional graphs, see Appendix A). Hence, to examine this, we proceeded to non-parametric Kruskal–Wallis tests to ascertain any potential differences between the two groups in the different times of measurement. The subsequent results are presented below. 

### 3.3. Group Differences at Each Time of Measurement after the Training Sessions

The Kruskal–Wallis test, a non-parametric approach, was utilized to compare the Experimental and Control groups at post-training and 3-month and 6-month follow-up measurements. The group was identified as the independent variable, while each cognitive and metacognitive outcome was defined as a dependent variable. The Kruskal–Wallis test can be applied separately to each time point. This means that even if a participant’s data are missing for some time points, their data from other time points can still be used in the analysis. Given its compatibility with small sample sizes, the Kruskal–Wallis test was considered suitable for our study. 

#### 3.3.1. Post-Training

**Figure 6:**
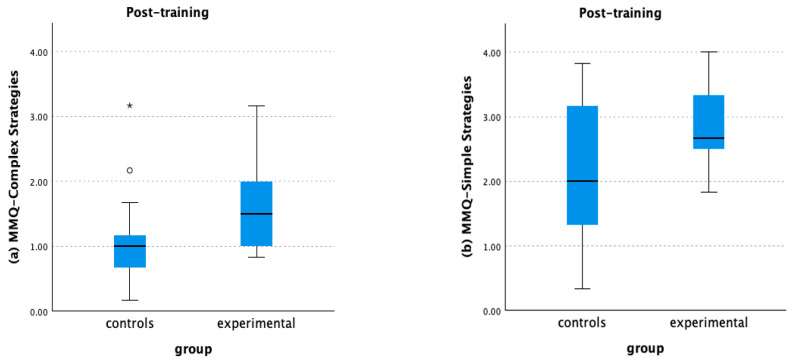
Experimental group vs. Control group and use of strategies post-training.

Following the training, it was revealed that the EG used compensatory strategies more frequently than the CG. Specifically, there was a statistically significant difference in MMQ—Complex Strategies, H(1, 42) = 9.66, *p* = 0.002, between the rank scores of the EG (27.36) and the CG (15.64). Also, a significant difference was detected in MMQ—Simple Strategies, H(1, 42) = 4.38, *p* = 0.036, between the rank scores of the EG (25.45) and the CG (17.55) (see Figure 6). No other significant differences between the groups were found post-training.

#### 3.3.2. Three-Month Follow-Up

At the 3-month follow-up, the EG demonstrated superior performance to the CG in terms of the WCST’s score for perseverative responses, H(1, 43) = 4.33, *p* = 0.038. Group differences were also significant in metacognitive outcomes. The EG demonstrated superior monitoring accuracy in DnP—Doors in comparison to the CG, H(1, 43) = 4.43, *p* = 0.035. Additionally, the EG reported a higher frequency of complex strategy use, H(1, 43) = 6.12, *p* = 0.013, and improved metacognitive beliefs of efficacy regarding attention compared to the CG: MKEA—Divided and Shifted Attention, H(1, 43) = 6.85, *p* = 0.009, and MKEA—Concentration, H(1, 43) = 6.36, *p* = 0.012. No other significant group differences were detected at the 3-month follow-up. To facilitate a comprehensive review of these findings, the complete dataset is available in Table 4, and for a visual representation of the difference, refer to Graphs a-e (Appendix B).

#### 3.3.3. Six-Month Follow-Up

Six months after training, the EG showed significant improvement over the CG. This was evident in the cognitive outcomes, such as the WCST scores for total correct, H(1, 43) = 7.19, *p* = 0.007, perseverative errors, H(1, 43) = 7.69, *p* = 0.006, and perseverative responses, H(1, 43) = 10.75, *p* = 0.001, as well as the DnP results for immediate figure recall, H(1, 43) = 4.05, *p* = 0.044. 

Similarly, there were significant differences in the metacognitive control outcomes between the two groups. Upon an initial examination of the overall scores for each variable for metacognitive control, as described in the mixed ANOVAs, we identified significant differences between groups. These were evident in the overall monitoring accuracy, H(1, 43) = 4.89, *p* = 0.027, overall global monitoring, H(1, 43) = 7.42, *p* = 0.006, and overall ‘wrong yes’ responses, H(1, 43) = 9.70, *p* = 0.002. Then, we conducted a further exploration of online metacognitive variables for each test. Thus, upon closer examination, we found that the EG demonstrated significantly better outcomes than the CG in online metacognitive measures associated with the WCST: monitoring accuracy, H(1, 43) = 11.05, *p* < 0.001, global monitoring, H(1, 43) = 9.12, *p* = 0.003, and ‘wrong yes’ responses, H(1, 43) = 11.59, *p* < 0.001. Furthermore, a significant difference was detected regarding the frequency of simple strategy use, where the EG reported a higher frequency compared to the CG, H(1, 43) = 4.10, *p* = 0.043. To facilitate a comprehensive review of these findings, the complete dataset is available in Table 5, while the differences are visually depicted in Graphs a–k (Appendix C).

## 4. Discussion

The aim of the present study was to longitudinally assess the efficacy of an MTP in people with aMCI. To do so, cognitive and metacognitive outcomes were compared between two groups—Experimental and Control—at four different time points—before–after–3 months–6 months after intervention. Additionally, based on recent findings indicating that individuals with MCI often struggle to accurately monitor their performance during active cognitive processes, we aimed to determine whether the MTP could yield sustained positive effects on online metacognitive outcomes over time.

Regarding cognitive outcomes, the results revealed that both the CG and the EG showed improvements after the training sessions in the memory and the executive functions tasks. However, significant differences emerged between the two groups at the 3-month follow-up, with the EG making fewer perseverative responses on the WCST compared to the CG. The differences became more pronounced at the 6-month follow-up, where the EG outperformed the CG in the WCST scores for total correct responses, perseverative errors, and perseverative responses indicating that the applied MTP bolsters cognitive flexibility and adaptability to new information over time. Additionally, superior performance was observed in the EG regarding immediate visual recall, as measured by the DnP Figures test. Therefore, while both—cognitive and metacognitive—training programs showed post-training benefits in cognition, the improvements for the CG began to wane after three months of the training’s completion. In contrast, participants in the EG not only preserved their gains but continued to show improvement, as indicated by the observed trends. 

Notably, the MTP exhibited specific benefits on cognitive flexibility, an area where individuals with MCI also show deficiencies [87,88,89]. As a core aspect of executive functions, cognitive flexibility plays an essential role in metacognitive regulation [90]. It describes an individual’s ability to recognize when their current cognitive strategies are not leading to the intended cognitive outcome. Instead of persistently utilizing ineffective methods, cognitive flexibility facilitates a shift from the current strategy, enabling the adoption of alternative approaches to optimize learning outcomes [72]. The observed enhancement in cognitive flexibility following the MTP may be largely attributed to its emphasis on enhancing metacognitive monitoring skills. Through consistent training, participants seemingly developed a heightened self-awareness of their ongoing strategies. When confronted with inefficacy, they were primed to recognize and adjust. This is in line with [91] which underscores the synergy between cognitive flexibility and the metacognitive monitoring processes. 

Similarly, regarding the online metacognitive outcomes following the interventions, both groups showed improvements in their online metacognitive scores compared to pre-training. Specifically, improvements were detected in metacognitive control as participants in both groups became more precise in deciding which responses to submit. These improvements may have been influenced by the design of the testing sessions, where participants were asked to report their confidence in each response and determine whether to include each response in their final score. Previous studies have shown that older adults without cognitive deterioration [92,93] and people with MCI [20] can improve their metacognitive accuracy through task-related experience. This could offer an additional explanation as to why the CG also demonstrated improvements in online metacognitive measures. 

Nonetheless, although at post-training the EG and CG showed improvements, a different course of the trend emerged at the 3-month follow-up between the two groups. Particularly, at the 3-month follow-up, the EG showed significantly higher monitoring accuracy in DnP—Doors compared to the CG. Furthermore, at the 6-month follow-up, these gains were reflected in improved scores for monitoring accuracy, global monitoring, and “wrong yes” in the WCST for the EG compared to the CG. These results emphasize the beneficial effects of MTP on metacognitive control. More specifically, the EG demonstrated enhanced precision in volunteering correct responses and withholding incorrect ones, compared to those in the CG. Considering the previously discussed connection between cognitive flexibility and metacognition, it is not surprising that these enhancements were more noticeable in the WCST. Therefore, the MTP promotes metacognitive control in individuals with MCI and the related gains are maintained over time.

Concerning metacognitive monitoring, participants from both groups exhibited improvements in their metacognitive ability scores over time. While no significant differences were detected between the two groups, a trend was also evident for this measure. This trend highlighted that individuals in the EG consistently displayed greater alignment between their confidence ratings and actual performance, whereas the CG showed a slight decline in this measure six months after training (refer to Figure 5).

The positive impact of the MTP was also detected in the offline metacognitive measures. Specifically, at the 3-month follow-up, participants in the EG reported a more frequent use of complex cognitive strategies and at the 6-month follow-up, a more frequent use of simple cognitive strategies than those in the CG. Interestingly, as time passed, EG participants demonstrated a preference for simple cognitive strategies over complex ones. Figure 6 graphically represents the declining trend in the use of complex strategies. Meanwhile, the upward trend for simple strategies is depicted in Graph (g) (see Appendix A for a visual representation). Although complex strategies, such as mental imagery, paired associations, and story creation, are effective in enhancing long-term memory due to their in-depth processing of information, they also require a greater investment of time and effort, making them potentially less practical for daily use. However, this finding is not necessarily a limitation. Throughout the training, even though the efficacy of these complex strategies was emphasized, we did not mandate their exclusive use. Instead, the purpose was to provide participants with new cognitive tools for use at their convenience. The primary focus of the training was to improve a more effective approach to cognitive tasks, underscoring the importance of investing time and effort to process information, recruit monitoring and control processes, and make necessary adjustments to achieve the intended outcome. We believe the MTP effectively served this purpose. 

Furthermore, improvements in self-efficacy were also detected. Individuals in the EG at the 3-month follow-up reported higher beliefs of efficacy regarding daily life scenarios related to attention compared to the CG. Memory self-efficacy also saw a subtle rise, interestingly, in both groups. However, no changes were observed in metacognitive beliefs about everyday executive functions. This outcome was anticipated given the already high scores both groups reported on this scale. The improved metacognitive beliefs concerning attention align with the principles of the MTP, as explained in the previous paragraph. Since the EG was trained in using and enhancing self-regulatory skills, with attention being pivotal in these processes, participants were specifically instructed on the significance of shifting and focusing their attention on the information of interest. This guidance likely fostered the better control and monitoring of attention processes, eventually leading to higher self-perceived efficacy in this aspect.

Overall, the results confirmed our initial hypothesis suggesting the sustained effects of the MTP in cognitive and metacognitive measures over a period of six months. Already, three months post-training, the CG began to show a decline in training-related gains. In contrast, the EG’s performance consistently improved, highlighting the superior efficacy of the MTP. Even though the effects observed at six months post-training might be seen as medium-term rather than long-term, we believe our findings are a significant contribution to the research on MTP efficacy. Remarkably, given the brief nature of the MTP—just ten sessions—it is impressive that individuals with MCI were not only able to gain benefits but also sustain progress over six months, especially considering their challenges in acquiring new information. However, future research should certainly consider incorporating assessments at longer intervals, such as beyond a year, to effectively capture these extended effects. We believe that the individualized format of the MTP further enhanced these positive outcomes since an individualized approach tailors each participant’s needs. This enables a more targeted intervention [39,45]. The clinical implications of this type of training are wide-ranging, as it can be tailored to the specific needs of various clinical populations. Accordingly, it would be valuable for clinical practice if future studies attempt to establish a specific protocol for designing and implementing an MTP, which clinicians can then adjust to the specific needs of each clinical population.

Furthermore, the current study introduces an innovative approach by pioneering the exploration of the effects of the MTP on online metacognitive measures—a subject yet to be thoroughly investigated. This exploration is crucial, as prior studies have indicated that deficiencies in metacognitive skills during cognitive tasks can lead to poorer outcomes and ineffective strategies when dealing with cognitive challenges [23,24,25,26]. Based on our findings, participants in the MTP showed improvement in their monitoring and metacognitive control skills. Specifically, they made more accurate decisions regarding which answers to volunteer and which to withhold. They also increased the use of compensatory cognitive strategies. It would be very informative for future studies to integrate neuroimaging data to understand how these changes manifest at the neural level. For example, they might observe increased functional connectivity within the frontoparietal network, which is pivotal for self-regulatory and control processes [94].

## 5. Limitations

Certainly, this study has some important limitations that should be acknowledged. First, the small sample size limited the statistical power of the findings. To mitigate this effect, we conducted a very thorough statistical analysis, which is extensively presented in the present paper. Second, the study’s design was single-blind, meaning the researcher conducting the testing and training sessions was aware of the group to which each participant was allocated. To minimize this potential bias, the researcher was dedicated to fully complying with and strictly applying the randomization procedure. Although the researcher maintained a strict level of professionalism and impartiality, the possibility for unintended bias cannot be completely excluded. Furthermore, the research was conducted during strict lockdown measures in order to deal with the COVID-19 pandemic. Under these conditions, the entire procedure had to be carried out online, and this may have compromised the integrity of the testing procedures. In addition, due to the imposed lockdown, participants had limited opportunities to apply cognitive strategies in everyday life. Also, existing research has highlighted the negative cognitive effects of prolonged lockdown [95,96]. Thus, the observed benefits might have been more significant under normal circumstances. Moreover, although the measurements and follow-up time employed in this study hold significance, it is crucial to track these data for a minimum period of 3 to 5 years to determine the long-term effects or to examine the impact of the MTP on MCI from diverse pathologies and subtypes [28,97]. 

Future studies should aim to overcome the aforementioned limitations. Additionally, researchers could explore several avenues for further investigation. Firstly, the integration of neuroimaging data could provide invaluable insights into the neural impact of metacognitive training. Secondly, the use of sensitive neuropsychological tests may reveal clearer patterns or differentiation between healthy individuals and those with MCI, addressing concerns about the limited sensitivity and specificity of the WCST [98]. Lastly, it would be beneficial to investigate the potential necessity for booster sessions to sustain training gains over time, including determining their optimal frequency.

## 6. Conclusions

Beside the limitations that this study holds, it provided novel insight into the application and efficacy of the MTP in addressing cognitive decline, particularly in individuals with MCI. The sustained effects observed over a six-month period underscore the potential of the MTP to significantly impact cognitive and metacognitive functioning, offering promising avenues for intervention in clinical settings. Given these findings, future research endeavors should prioritize investigations aimed at elucidating the mechanisms underlying the observed effects and expanding upon the MTP’s potential applications in diverse clinical contexts.

## Figures and Tables

**Table 1:** Metacognitive training program: overview of each session’s specific content and homework assignments.

Session	Content	Homework
Introduction	The aim of the metacognitive training program (MTP). Introduction to cognitive functions. An exploration of their basic domains. Discussion on factors that affect cognitive functions. The importance of understanding these processes.	To answer the following questions: “How often do I forget to do something?” “What can I do to remember better (think about what we’ve said: factors that affect cognitive functions, cognitive functions affect each other)?” “Do I use any strategy or aid?” “If yes, which one(s)?”
Memory I	Introduction to memory function and subsystems: Memory stages; Short-term memory; Long-term memory.	A set of exercises with lists to learn. Immediate recall and delayed recall. Instructions for self-testing. Answering questions such as giving a brief description of the techniques used to learn the required material or suggesting what could have been performed differently to enhance performance.
Memory II	Memory subsystems II: Episodic and autobiographical memory; Prospective memory; Semantic memory; Procedural memory.	A set of exercises with different types of material to learn (story, lists, numbers) including immediate and delayed recall. Instructions for self-testing. Answering to questions such as giving a brief description of the techniques used to learn the required material or suggesting what could have been performed differently to enhance performance.
Attention	Divisions of attention: Selective attention; Sustained attention; Divided attention; Shifted attention. Stress and attention	A set of exercises targeting the different dimensions of attention that mirror real-world situations. Responding to questions about the perceived level of difficulty encountered during the exercise’s completion, and if any difficulties were faced, what those challenges were.
Executive Functions	Executive functions and their role in everyday life. Aspects of executive functions: Decision-making and inhibition; Problem-solving and flexible thinking; Planning.	A set of exercises targeting the planning of daily activities and problem-solving tasks that mirror real-world situations. Responding to questions about the perceived level of difficulty encountered during the exercise’s completion, and if any difficulties were faced, what those challenges were.
Aging	Aspects of aging: cognitive, social, physical, and psychological. Positive and negative consequences. Mild cognitive impairment: myths and truths.	A small essay based on 3 questions: What aspects of my life are better now compared to 20 or 30 years ago? Based on my current understanding of cognitive functions, where do I identify challenges? Provide specific examples from your everyday life. What changes can I make in my life to improve my cognitive functions? Provide specific examples from your everyday life.
Cognitive Strategies: Introduction	Introduction to different types of cognitive strategies: external and internal. Understanding the significance of using cognitive strategies. Showcase and training on strategies: categorization, verbal and visual association, story creation, and mental imagery.	The provision of diverse exercises to practice cognitive strategies, covering different types of information: lists of words or pictures, stories, a program of daily activities, or numbers. Encouragement to use any chosen strategy from the presented options, with the stipulation that each strategy should be used at least once. An evaluation of the effectiveness of the implemented strategies. Encouragement to consider alternate daily situations where each strategy could be effectively applied. The distribution of a “strategy-diary” for participants to record when and what strategy they applied in their daily life during the week.
Cognitive Strategies I	Q&A about cognitive strategies and the related homework. Further practice.	
Cognitive Strategies II	Q&A about cognitive strategies and the related homework. Further practice.	
Cognitive Strategies: Closing	Q&A about cognitive strategies and the related homework. Further practice. Overall discussion and encouragement to keep using cognitive strategies.	

**Table 2:** Participants’ demographic characteristics.

	EG ^a^ (*n* = 22)		CG ^b^ (*n* = 23)			
	Mean	SD ^c^	Mean	SD	F	*p* ^d^
Demographics						
Age	63.27	5.62	62.30	6.88	0.266	n.s. ^e^
Education	13.59	3.76	12.65	3.14	0.828	n.s.
Gender (f/m)	14/8		16/7		χ^2^	n.s.

Note. ^a^ EG = Experimental group received metacognitive training. ^b^ CG = Control group received cognitive training. ^c^ SD = standard deviation. ^d^ *p* < 0.05. ^e^ n.s. = non-significant.

**Table 3:** Cognitive and metacognitive outcomes between Control and Experimental groups at different time points.

			Mean Difference	Standard Error	Sig.	95% Confidence Interval for Difference	
						Lower Bound	Upper Bound
WCST: ^a^ Total Correct	Pre-training	Post-training	−2.60	1.61	n.s. ^b^	−7.09	1.89
		3m follow-up	−4.40	1.50	n.s.	−8.57	−0.23
		6m follow-up	−5.23	1.49	0.01	−9.38	−1.08
WCST: Total Errors	Pre-training	Post-training	2.76	1.62	n.s.	−1.76	7.27
		3m follow-up	4.71	1.52	n.s.	0.48	8.95
		6m follow-up	5.67	1.49	0.00	1.53	9.82
WCST: Non-Perseverative Errors	Pre-training	Post-training	3.04	1.17	n.s.	−0.23	6.31
		3m follow-up	4.36	1.15	0.00	1.17	7.55
		6m follow-up	5.91	1.09	0.00	2.89	8.94
WCST: Categories	Pre-training	Post-training	−0.44	0.25	n.s.	−1.14	0.27
		3m follow-up	−0.63	0.27	n.s.	−1.37	0.11
		6m follow-up	−0.82	0.26	0.02	−1.53	−0.10
DnP ^c^—People: Immediate Verbal Recall	Pre-training	Post-training	−3.81	1.00	0.00	−6.60	−1.02
		3m follow-up	−6.20	1.04	0.00	−9.10	−3.30
		6m follow-up	−6.20	0.99	0.00	−8.95	−3.45
DnP—Figures: Immediate Visual Recall	Pre-training	Post-training	−1.61	0.53	0.02	−3.08	−0.15
		3m follow-up	−1.82	0.56	0.01	−3.39	−0.26
		6m follow-up	−2.24	0.74	0.03	−4.32	−0.17
DnP—Doors: Visual Recognition	Pre-training	Post-training	−1.81	0.37	0.00	−2.83	−0.78
		3m follow-up	−2.57	0.36	0.00	−3.58	−1.56
		6m follow-up	−2.93	0.43	0.00	−4.11	−1.74
DnP—Names: Verbal Recognition	Pre-training	Post-training	−1.29	0.51	n.s.	−2.72	0.14
		3m follow-up	−1.72	0.51	0.01	−3.15	−0.29
		6m follow-up	−2.25	0.44	0.00	−3.48	−1.03
DnP—People: Delayed Verbal Recall	Pre-training	Post-training	−0.52	0.19	n.s.	−1.05	0.02
		3m follow-up	−0.65	0.17	0.00	−1.13	−0.16
		6m follow-up	−0.84	0.20	0.00	−1.40	−0.29
Monitoring Accuracy	Pre-training	Post-training	−0.08	0.02	0.01	−0.14	−0.01
		3m follow-up	−0.10	0.02	0.00	−0.15	−0.05
		6m follow-up	−0.11	0.02	0.00	−0.15	−0.06
Global Monitoring	Pre-training	Post-training	−0.70	0.37	n.s.	−1.73	0.34
		3m follow-up	−1.37	0.38	0.01	−2.43	−0.31
		6m follow-up	−1.34	0.35	0.00	−2.31	−0.36
Wrong Yes	Pre-training	Post-training	0.91	0.28	0.01	0.13	1.69
		3m follow-up	1.45	0.23	0.00	0.81	2.09
		6m follow-up	1.60	0.27	0.00	0.84	2.37
Feeling of Confidence	Pre-training	Post-training	−0.25	0.05	0.00	−0.38	−0.11
		3m follow-up	−0.28	0.04	0.00	−0.39	−0.16
		6m follow-up	−0.31	0.05	0.00	−0.43	−0.18
MMQ ^d^: Complex Strategies	Pre-training	Post-training	−0.53	0.13	0.00	−0.89	−0.17
		3m follow-up	−0.45	0.10	0.00	−0.72	−0.17
		6m follow-up	−0.49	0.11	0.00	−0.78	−0.19

Note. ^a^ WCST = Wisconsin Card Sorting Test. ^b^ n.s. = non-significant. ^c^ DnP = Doors and People. ^d^ MMQ = Multifactorial Metamemory Questionnaire.

**Table 4:** Group differences at 3-month follow-up.

Outcome	Group EG ^a^ (*n* = 22) vs. CG ^b^ (*n* = 21)	Mean Rank	χ^2^	*p*-Value
WCST ^c^: Perseverative Responses	Experimental Group	26	4.33	0.038
	Control Group	18.18		
Monitoring Accuracy: DnP ^d^—Doors	Experimental Group	25.93	4.43	0.035
	Control Group	17.88		
MMQ ^e^—Complex Strategies	Experimental Group	26.61	6.12	0.013
	Control Group	17.17		
MKEA ^f^: Divided and Shifted Attention	Experimental Group	26.84	6.85	0.009
	Control Group	16.93		
MKEA: Concentration	Experimental Group	26.66	6.36	0.012
	Control Group	17.12		

Note. ^a^ EG = Experimental group. ^b^ CG = Control group. ^c^ WCST = Wisconsin Card Sorting Test. ^d^ DnP = Doors and People. ^e^ MMQ = Multifactorial Metamemory Questionnaire. ^f^ MKEA = Metacognitive Knowledge of Everyday Attention.

**Table 5:** Group differences at 6-month follow-up.

Outcome	Group EG ^a^ (*n* = 21) vs. CG ^b^ (*n* = 22)	Mean Rank	χ^2^	*p*-Value
WCST ^c^: Total Correct	Experimental Group	27.21	7.19	0.007
	Control Group	17.02		
WCST: Perseverative Errors	Experimental Group	16.62	7.69	0.006
	Control Group	27.14		
WCST: Perseverative Responses	Experimental Group	15.76	10.75	0.001
	Control Group	27.95		
DnP ^d^—Figures I: Immediate Visual Recall	Experimental Group	25.48	4.05	0.044
	Control Group	18.68		
Overall Monitoring Accuracy	Experimental Group	26.33	4.89	0.027
	Control Group	17.86		
Overall Global Monitoring	Experimental Group	27.33	7.42	0.006
	Control Group	16.91		
Overall Wrong Yes	Experimental Group	27.82	9.70	0.002
	Control Group	15.90		
Monitoring Accuracy: WCST	Experimental Group	28.50	11.05	<0.001
	Control Group	15.80		
Global Monitoring: WCST	Experimental Group	27.90	9.12	0.003
	Control Group	16.36		
Wrong Yes: WCST	Experimental Group	15.38	11.59	<0.001
	Control Group	28.32		
MMQ ^e^—Simple Strategies	Experimental Group	25.95	4.10	0.043
	Control Group	18.23		

Note. ^a^ EG = Experimental group. ^b^ CG = Control group. ^c^ WCST = Wisconsin Card Sorting Test. ^d^ DnP = Doors and People. ^e^ MMQ = Multifactorial Metamemory Questionnaire.

## Data Availability

The data of this study are available at DOI: 10.17632/s24bb5p42t.1.

## Appendix A

Additional graphs are presented below that offer a visualization of the observed trend between the Experimental and the Control group in variables that did not reveal statistically significant differences between the two groups.

## Appendix B

These plots offer a visual demonstration of the differences between the Experimental and the Control group at the 3-month follow-up.

## Appendix C

These plots offer a visual demonstration of the differences between the Experimental and the Control group at the 6-month follow-up.
